# Improved surgical outcomes and predictors of prolonged hospitalization in descending necrotizing mediastinitis: A 20-Year retrospective study

**DOI:** 10.1007/s11748-026-02261-3

**Published:** 2026-02-12

**Authors:** Masako Chiyo, Takekazu Iwata, Takamasa Ito, Yuki Sata, Takahide Toyoda, Terunaga Inage, Kazuhisa Tanaka, Junichi Morimoto, Yukiko Matsui, Hidemi Suzuki

**Affiliations:** 1 Department of General Thoracic Surgery, Chiba University Graduate School of Medicine, 1-8-1, Inohana,chuo-ku, Chiba, 260-8670 Japan; 2 Department of Thoracic Surgery, Chiba cancer center, Chiba, Japan; 3 Department of Thoracic Surgery, National Hospital Organization Chiba Medical Center, Chiba, Japan

**Keywords:** Descending necrotizing mediastinitis (DNM), Surgical debridement, Video-assisted thoracoscopic surgery (VATS), Endo classification

## Abstract

**Objective:**

Descending necrotizing mediastinitis (DNM) is a life-threatening infection that causes by cervical infections. An appropriate diagnosis and surgery are critical for improving outcomes. We retrospectively analyzed 37 surgically treated patients over a 20-year period to identify factors associated with improved survival and prolonged hospitalization.

**Methods:**

Patients who underwent combined cervical and thoracic drainage for DNM at our institution in 2004–2024 were reviewed. Clinical data, surgical approaches, outcomes, and risk factors for long-term hospitalization were analyzed.

**Results:**

The cohort (*n* = 37) included 22 males (59.5%), with a mean age of 64.1 years. Endo’s classification was type IIB (extension to the lower posterior mediastinum) in 28 cases. Bilateral thoracotomy (including VATS) was performed in 23 patients (mean time from onset to surgery: 5.8 days). The median ICU and hospital stays was 20.9 and 59.3 days, respectively. The 90-day mortality rate was 8.1% (*n* = 3). All non-survivors were elderly and had renal impairment at admission. Among 34 survivors, a multivariate analysis identified age (*p* = 0.0012), odontogenic origin (*p* = 0.0305), and drainage duration (*p* = 0.0047) as independent predictors of prolonged hospitalization. Notably, 72.2% of short-stay patients were treated within the last 10 years, indicating improved outcomes.

**Conclusion:**

*Our 20-year experience suggests elderly patients with renal impairment were at high risk of early mortality. In survivors*,* age*,* odontogenic origin*,* and drainage duration predicted prolonged hospitalization.* Future efforts should focus on minimizing the hospitalization duration while maintaining effective infection control.

## Introduction

Descending necrotizing mediastinitis (DNM) is a rare but life-threatening form of acute mediastinitis that typically arises as a complication of odontogenic or cervicofacial infection. Despite its low incidence, the rapid progression of infection and anatomical complexity of the mediastinum make DNM a challenging condition that requires a prompt diagnosis and multidisciplinary treatment [1].

We previously reported our experience with 10 cases treated between 1991 and 2003, advocating an aggressive treatment strategy that included broad-spectrum antibiotics, combined cervical and thoracic debridement, and postoperative mediastinal irrigation [2]. Since then, DNM management has evolved, supported by classification systems and improved imaging-based surgical decision-making. In particular, Endo et al. proposed a CT-based classification of DNM that stratifies disease extent and recommends tailored surgical approaches accordingly [3]. Recently, Sugio et al. updated this framework and emphasized its relevance to modern treatment strategies in Japan [4].

Minimally invasive approaches such as video-assisted thoracoscopic surgery (VATS) have gained attention for reducing surgical invasiveness while maintaining efficacy with favorable outcomes reported in selected cases [5]. However, open thoracotomy continues to play an important role in extensive disease, as highlighted in earlier reports emphasizing the need for aggressive surgical intervention [6]. Improvements in imaging, early diagnosis, and coordinated care have contributed to declining mortality rates, with some centers achieving substantial improvements in survival in recent years [7]. To address this issue, we retrospectively reviewed 20 years of cases, including 37 patients who underwent surgery for DNM at our department.

The objective of this study was to identify the clinical factors associated with mortality and to determine predictors of prolonged hospitalization in patients with DNM. These findings may inform strategies to improve outcomes and reduce postoperative complications in DNM patients. We aimed to examine not only predictors of mortality, but also factors contributing to prolonged hospitalization, which has become an increasingly important clinical concern in aging populations.

## Subjects

We reviewed the medical and surgical records of all 37 patients who underwent surgical treatment for DNM at the Department of General Thoracic Surgery, Chiba University Hospital, between January 2004 and December 2024 by referring to medical records. The extent of DNM was classified according to the Endo classification, which categorizes infection based on its mediastinal spread into Type I (localized infection limited to the area superior to the carina level), IIA (infection extending to the lower anterior mediastinum), and IIB (infection spread to the lower posterior mediastinum) [3]. Cervical and mediastinal drainage and debridement were performed collaboratively by the Thoracic Surgery and Otolaryngology teams. In this study, procedures were classified as VATS when performed through an incision of ≤ 8 cm under thoracoscopic vision, provided that adequate drainage was achieved without conversion. As a result, the proportion of VATS increased in later years. Open thoracotomy was defined as any larger incision with direct exposure. The choice between unilateral and bilateral thoracotomy was guided by CT: when collections were judged drainable from one side, a unilateral approach was used; if bilateral mediastinal spread or pleural involvement was present, or contralateral purulence was found intraoperatively, bilateral thoracotomy was performed. Following surgery, all patients were admitted to the intensive care unit (ICU) for at least one day for intensive monitoring and systemic management, and all survivors received postoperative rehabilitation and nutritional support during hospitalization.

### Methods

Patients were categorized into two groups for analysis. First, a comparison was made between survivors(*n* = 34) and non-survivors(*n* = 3). A comparative analysis was performed between survivors (*n* = 34) and non-survivors (*n* = 3). Among the survivors, patients were further divided into two groups based on the median hospital stay (52 days): the short-term group (≤ 52 days) and the long-term group (> 52 days). To identify factors associated with prolonged hospitalization, univariate and multivariate analyses were performed between these two groups.

### Statistical analysis

Continuous variables were presented as means with standard deviations, and categorical variables were presented as frequencies with percentages. Categorical variables were compared using Pearson’s chi-squared test, whereas continuous variables were compared using Student’s t-test. All tests were two tailed. Statistical significance was set at *p* < 0.05. Statistical analyses were performed using JMP (ver. 18, SAS Institute, Cary, NC, USA).

This retrospective study was based on previously collected medical records and approved by the Ethics Committee of Chiba University Hospital (approval no. HK202501-07). As obtaining informed consent from individual patients is impractical, the study protocol was approved using an opt-out method.

## Results

During the study period, 37 patients underwent combined thoracic and otolaryngological surgeries for DNM. The patient characteristics are listed in Tables 1 and 2. There were 22 males and 15 females with a mean age of 64.1 years. According to the Endo classification, 5 cases were type I, 4 were type IIA, and 28 were type IIB. The sources of infection included pharyngitis or pharyngeal abscess in 16 patients (43.2%), odontogenic infections in 5 patients (13.5%), and other cervical abscesses in the remaining 16 patients. The most common comorbidities were diabetes mellitus (29.7%) and hypertension (21.6%). Anaerobic bacteria were identified as causative pathogens before surgical intervention in 65% of cases. Preoperative data showed that the mean white blood cell count was 11,230, the mean C-reactive protein level was 29, and that 40.5% of patients had renal impairment at admission. Serum albumin levels at admission were markedly low (mean 2.2 g/dL). The surgical techniques employed included bilateral thoracotomy (including VATS) in 23 cases, unilateral thoracotomy in 11 cases, and anterior superior mediastinal drainage in three cases. In addition, tracheostomy was required in 34 patients (91.8%) for prolonged airway management. On average, the time from the onset of symptoms to surgery was 5.7 days. Reoperations, including additional drainage for newly formed abscesses, were performed in 7 patients (3 in the long-term group and 4 in the short-term group). Reoperation did not show a significant association with the length of hospitalization. The median ICU and hospital stays were 20.9 and 59.3 days, respectively. The 90-day mortality rate 8% (*n* = 3), including two cases of sepsis and one case of cerebral hemorrhage due to meningitis. All patients were in their 70s and had renal impairment at the time of presentation. Notably, there were no deaths after 2008.

**Table 1:** Patient characteristics

Variables	*n* = 37	
Age (range)	62.7 ± 11.7 (25–88)	
Sex		
Male (%)	22 (59.5%)	
Female (%)	15 (40.5%)	
Cause of disease (source of infection)		
Pharyngeal (%)	16 (43.2%)	
Odontogenic (%)	5 (13.5%)	
Other cervical (%)	16 (43.2%)	
Type of DNM (Endo classification)		
Type I (%)	5 (13.5%)	
Type IIA (%)	4 (10.8%)	
Type IIB (%)	28 (75.7%)	
Comorbidity		
DM	11 (29.7%)	
HT	8 (21.6%)	
Preoperative data		
White blood cell (/µL)	11230 ± 6924	
C-reactive protein (mg/dL)	29 ± 14	
Serum albumin (g/dL)	2.2 ± 0.6	
Renal impairment at admission (% of coexistence)	15 (40.5%)	
Anaerobic bacteria (%)	24 (65%)	
Interval from onset to thoracic surgery (days)	5.7 ± 3.8	
Interval from hospitalization to thoracic surgery	1.0 ± 0.2	

DNM, descending necrotizing mediastinitis; DM, diabetes mellitus; HT, hypertension;

**Table 2:** Treatment and outcomes

Variables	*n* = 37
Surgical method	
Bilateral Thoracotomy (%)	23 (62.2%)
Unilateral thoracotomy (%)	11 (29.7%)
Mediastinum drainage via subxiphoid (%)	3 (8.1%)
Approach for mediastinal drainage	
VATS (%)	7 (18.9%)
Thoracotomy (%)	27 (73.0%)
Subxiphoid (%)	3 (8.1%)
Tracheostomy (%)	34 (91.8%)
Number of chest tubes	5.8 ± 2.6
Duration of drainage (days)	17.9 ± 9.7
Hospital stay (days from surgery)	59.3 ± 42.9
ICU stay (days from surgery)	20.9 ± 16.0
90-day survival rate (%)	34 (91.8%)

DNM, descending necrotizing mediastinitis; VATS, video-assisted thoracic surgery

Table 3 compares the clinical characteristics and outcomes of patients who died in the hospital (*n* = 3) and those who survived (*n* = 34). The non-survivors were significantly older than the survivors (mean age: 74.0 vs. 63.2 years, *p* = 0.0446). Renal impairment indicated by elevated serum creatinine (> 1.5 mg/dL) on admission was observed in all three non-survivors, which was significantly more frequent than that in survivors (100% vs. 35.3%, *p* = 0.0287). Additionally, the preoperative white blood cell counts of non-survivors were significantly lower in comparison to survivors (5033/µL vs. 14679/µL, *p* = 0.0369). However, CRP and serum albumin were not significantly associated with survival. Although not statistically significant, there were trends indicating that non-survivors presented with Endo type I disease, lacked an odontogenic origin, and underwent earlier thoracic surgery (mean interval from onset to surgery: 2.3 vs. 6.1 days, *p* = 0.0324). These findings imply that a poor baseline condition, rather than disease extent or delay in surgery, may have been the key determinant of the outcome of these cases.

**Table 3:** Comparison of survivors and non-survivors (hospital death)

Variables	Non-survivors (*n* = 3)	Survivors (*n* = 34)	*p*-value
Age (year)	74.0 ± 6.6	63.2 ± 1.9	0.0446*
Sex (% of male)	33.3%	61.8%	0.5541
Cause of disease (Pharyngeal/Odontogenic/Other cervical)	3/0/0	13/5/16	0.1117
DNM-Endo classification (I/IIA/IIB)	2/0/1	3/4/27	0.0808*
DM (% of coexistence)	66.7%	26.4%	0.1442
HT (% of coexistence)	0%	23.5%	0.3426
Renal impaiment at admission	100%	35.3%	0.0287*
Surgical method (bilateral/unilateral/subxiphoidal)	1/2/0	22/9/3	0.3330
Approach for mediastinal drainage (VATS/ thoracotomy /subxiphoidal)	0/3/0	7/24/3	0.5463
Preoperative white blood cells (/µL)	5033.3 ± 3189.6	14679.4 ± 9011.8	0.0369*
Preoperative C-reactive protein (mg/dL)	31.2 ± 20.6	29.5 ± 10.7	0.6764
Serum albumin (g/dL)	2.7 ± 0.3	2.2 ± 0.1	0.1062
Anaerobic bacteria (%)	33.3%	67.7%	0.2777
Interval from onset to thoracic surgery	2.3 ± 2.2	6.1 ± 0.7	0.0324
Interval from hospitalization to thoracic surgery	0.3 ± 1.3	1.1 ± 0.4	0.448

DNM, descending necrotizing mediastinitis; DM, diabetes mellitus; HT, hypertension; VATS, video-assisted thoracic surgery. * *p* < 0.05

Moreover, among the 34 patients who survived DNM, we compared the clinical characteristics of those with long-term hospitalization (*n* = 16) to those with short-term hospitalization (*n* = 18) (Table 4). Patients in the long-term hospitalization group were significantly older (68.5 ± 2.7 vs. 58.4 ± 2.5 years, *p* = 0.0106). Specifically, cases of pharyngeal origin were more prevalent in the short-term hospitalization group, whereas those of odontogenic and cervical origins were more common in the long-term hospitalization group. Regarding the surgical strategy, bilateral thoracotomy tended to be more frequent in the short-term hospitalization group compared with the long-term group (72.2% vs. 68.8%), but the difference did not reach statistical significance (*p* = 0.0584, Table 4). Although not statistically significant, a subxiphoid approach tended to be used more frequently in the long-term group.

**Table 4:** Comparison of long-term hospitalization and short-term hospitalization among 34 survivors

Variables	Long-term hospitalization (*n* = 16)	Short-term hospitalization (*n* = 18)	*p*-value
Age (years)	68.5 ± 2.7	58.4 ± 2.5	0.0106*
Sex (% of male)	56.3%	66.6%	0.5327
Cause of disease (Pharyngeal/ Odontogenic/ Other cervical)	4/5/7	12/0/6	0.0112*
Type of DNM; Endo class I/IIA/IIB	1/3/12	2/1/15	0.4597
DM (% of coexistence)	37.5%	16.7%	0.1693
HT (% of coexistence)	22.2%	25%	0.8488
Renal impairment at admission	50%	22.2%	0.00907*
Interval from onset to thoracic surgery (days)	5.4 + 1.5	6.7 ± 0.9	0.3606
Interval from hospitalization to surgery (days)	1.2 ± 2.8	0.9 ± 1.9	0.8977
Preoperative white blood cells (/µL)	14344 ± 2155.3	15056 ± 2286	0.9039
Preoperative c-reactive protein (mg/dL)	27.4 ± 16.2	29.0 ± 11.8	0.790
Serum albumin (g/dL)	2.1 ± 0.1	2.3 ± 0.1	0.4765
Surgical method (bilateral/unilateral/subxiphoid)	11/2/3	11/7/0	0.0584
Approach for mediastinal drainage (VATS/ thoracotomy /subxiphoidal)	2/11/3	5/13/0	0.1136
Duration for drainage	22.4 ± 2.28	14.1 ± 2.15	0.0866

DNM, descending necrotizing mediastinitis; DM, diabetes mellitus; HT, hypertension; VATS, video-assisted thoracic surgery. *p < 0.05

No significant differences were observed between the groups in terms of sex distribution, Endo classification of DNM, presence of diabetes mellitus or hypertension, renal impairment, white blood cell count, preoperative C-reactive protein levels, serum albumin or time intervals from disease onset or hospital admission to surgery.

Table 5 shows univariable and multivariable logistic regression analyses to identify factors associated with prolonged hospitalization. In the univariable analysis, older age (odds ratio [OR], 1.11 per 1-year increase; 95% confidence interval [CI], 1.03–1.24; *p* < 0.001), odontogenic origin of disease (OR, 7.55; 95% CI, not specified; *p* < 0.001), and longer duration of drainage (OR, 1.11; 95% CI, 1.02–1.22; *p* = 0.011) were significantly associated with prolonged hospitalization.

**Table 5:** Univariate and multivariate analysis of factors for prolonged hospitalization

	Univariate analysis			Multivariate analysis
	OR	95%CI	*p*-value	*p*-value
Age per 1-year increase	1.11	1.03–1.24	< 0.001**	< 0.001**
Sex (Male/Female)	0.64	0.15–2.58	0.533	
Cause of disease (Odontogenic or not)	7.55	1.57-NA*	< 0.001**	0.031**
DM (yes/no)	3.00	0.64-17.0	0.167	
Renal impairment at admission	3.50	0.83–16.9	0.089	0.219
White blood cells (/µl) 1000 increase	1.01	0.93–1.09	0.816	
Interval from onset to thoracic surgery 1-day increase	0.92	0.75–1.09	0.342	
Surgical method (bilateral thoracotomy or not)	1.40	0.34–6.05	0.641	
Approach for mediastinal drainage (VATS or not)	0.37	0.047–2.06	0.264	
Tracheostomy	1.88	0.16–42.8	0.614	
Duration of drainage	1.11	1.02–1.22	0.011**	0.0047**

DM, diabetes mellitus; VATS, video-assisted thoracic surgery; OR odds ratio; CI, Confidence interval. *upper confident limit not calculable, **p < 0.05

In the multivariate analysis, age (adjusted *p* = 0.001), odontogenic origin (adjusted *p* = 0.031), and duration of drainage (adjusted *p* = 0.0047) remained independently associated with prolonged hospitalization. Other variables, such as sex, presence of diabetes mellitus or renal impairment, white blood cell count, interval from disease onset to thoracic surgery, surgical method (bilateral thoracotomy), surgical approach (video-assisted thoracic surgery [VATS]), and tracheostomy were not identified as significant factors in either analysis.

## Discussion

This 20-year retrospective analysis provides important insights into the management and outcomes of DNM. While advancements in diagnostic imaging and multidisciplinary care have contributed to improved survival in recent years, DNM remains a frequent morbidity, particularly in the elderly or patients with comorbidities. Our findings highlight not only the clinical factors associated with mortality but also suggest that prolonged hospitalization, often overlooked, represents a significant clinical and economic burden.

Timely and appropriate surgical drainage, antimicrobial therapy, and comprehensive systemic management are essential for improving survival, as emphasized in the relevant literature [8]. In addition to aggressive surgical drainage, advances in multidisciplinary care, including routine postoperative ICU management, rehabilitation support and intensive nutritional management, have likely contributed to the improvement in survival observed in recent years. However, the insidious progression of infection and anatomical complexity of the mediastinum require prompt and tailored interventions based on accurate imaging and clinical evaluation. It is encouraging to observe that awareness and understanding of DNM have significantly improved over the past two decades, resulting in better coordination and more effective care.

One of the key advantages of the present investigation was the extended duration of observation combined with a uniform treatment approach at a single institution. This consistency enabled the evaluation of temporal trends and identification of prognostic factors over two decades. Nevertheless, the retrospective and single-center design may limit the generalizability of our findings. In addition, certain host-related variables, such as nutritional status and frailty, were not systematically assessed and warrant further investigation in future prospective studies.

Previous studies have emphasized the importance of early and aggressive surgical drainage in DNM [8, 9]. Our findings support this, particularly for extensive infections classified as Endo types IIA and IIB, for which conservative management is often insufficient. Misthos et al. reported poor outcomes in conservatively managed type IIA cases [9], aligning with our experience. We found that drainage via the subxiphoid approach was effective in controlling infection but tended to prolong hospital stay, suggesting a trade-off between invasiveness and recovery speed. Accurate interpretation of CT findings and early selection of appropriate drainage methods, including unilateral or bilateral thoracotomy or VATS, are essential [10]. In our cohort, the mortality rate was approximately 8%, with three deaths occurring at 1, 56, and 84 days after surgery. Despite differences in causative organisms, these patients shared the following common characteristics: older age, pre-existing renal impairment, and low preoperative white blood cell counts. These findings are consistent with prior reports that highlight advanced age and renal dysfunction as poor prognostic factors for DNM [11–13]. Low white cell counts may reflect immunosenescence or septic exhaustion, particularly in elderly patients, leading to blunted inflammatory responses and poor infection control. Notably, no deaths have occurred in our department since 2008. We attribute this to improved referral systems and coordinated care by a multidisciplinary team, including otolaryngologists, thoracic surgeons, and intensivists.

Nevertheless, the need for tracheostomy and multiple drainage procedures have contributed to prolonged hospitalization. Neither bilateral thoracotomy nor the use of VATS showed a significant association with hospitalization length. Although reoperations were required in seven patients, all of whom survived, reoperation itself was not associated with longer hospitalization. This suggests that re-intervention for newly developed abscesses, when adequately performed, may not necessarily worsen the clinical course. These findings indicate that the choice of surgical method or approach, even with reoperation, once appropriate drainage is achieved, does not primarily determine the duration of hospitalization. Instead, drainage duration emerged as the critical determinant of hospital stay. Importantly, drainage duration was not dependent on the surgical method but likely reflected the underlying severity and persistence of infection. Thus, prolonged hospitalization in DNM appears to be driven more by disease biology and postoperative management needs than by surgical approach itself. Our cohort presented with severe malnutrition, with a mean serum albumin of 2.2 g/dL at admission. Although albumin was not directly associated with survival or length of hospitalization in this study, DNM is inherently a condition accompanied by profound nutritional decline and inevitably prolonged hospital stays. Even the ‘short-term’ hospitalization group required ≤ 52 days of hospitalization, underscoring the challenges of postoperative rehabilitation and nutritional management. In the analysis of 34 surviving patients, older age, odontogenic source of infection, and prolonged drainage duration were significantly associated with longer hospital stay. Odontogenic infections often involve deep fascial planes, are more difficult to access surgically, and require extensive drainage [13–15]. Additionally, unilateral or limited drainage (e.g., subxiphoid) was more common in patients with extended stays, whereas bilateral thoracotomy was more frequently associated with shorter hospitalization. These findings suggest that early, extensive drainage may facilitate infection resolution and earlier drain removal, thereby accelerating recovery and discharge planning [14, 15]. Importantly, prolonged hospitalization has significant implications for postoperative activities of daily living (ADLs) and healthcare costs. Reduced physical resilience in older patients slows recovery and increases the risk of long-term functional decline [16]. Furthermore, limited physical activity after discharge has been shown to be associated with persistent impairment, particularly in frail individuals [17]. While advancements in diagnostic imaging and multidisciplinary care have contributed to improved survival in recent years [4, 7], DNM remains a frequent morbidity, particularly in the elderly or patients with comorbidities [11, 12]. Ramos-Hinojosa et al. recently reported that elderly patients with deep neck abscess complicated by DNM admitted to the ICU had higher mortality and organ failure rates, highlighting the vulnerability of this population [18]. Similarly, Sugio et al. demonstrated improved outcomes with tailored surgical approaches in Japan, emphasizing the importance of timely diagnosis and coordinated multidisciplinary management [4.18]. Our findings are consistent with previous reports emphasizing the importance of early and aggressive drainage [3, 19]. Moreover, recent case reports have demonstrated the feasibility of innovative surgical approaches, such as VATS or combined cervical and thoracic strategies, in managing selected patients [20, 21].

Although DNM is rare, thoracic surgeons must be highly familiar with its clinical course and management. Prospective studies are difficult because of their rarity; therefore, the continuous accumulation of clinical experience and its critical evaluation remains essential. Future directions may include multicenter registries, refinement of risk stratification tools, and structured postoperative care protocols to reduce hospitalization and preserve the functional status of elderly patients.

## Conclusion

DNM is a rare, but life-threatening condition that often requires prompt multidisciplinary treatment, as emphasized in previous studies. Based on our 20-year experience, a poor baseline condition, rather than disease extent or delay in surgery, appears to play a critical role in outcomes of patients with DNM.　Notably older age, odontogenic origin, and prolonged drainage were associated with prolonged hospitalization. These findings highlight the importance of careful perioperative management aimed at optimizing patient condition and reducing hospital stay while maintaining effective infection control.
